# Acute Traumatic Tetanus Treated by Magnesium Sulphate

**Published:** 1909-03

**Authors:** Aime Paul Heineck

**Affiliations:** Chicago, Ill.; Professor of Surgery, Reliance Medical College; Adjunct Professor of Surgery, University of Illinois; Surgeon to the Cook Coutny Hospital


### ACUTE TRAUMATIC TETANUS TREATED BY MAGNESIUM SULPHATE. (continued).

WITH REPORT OF A CASE IN THE TREATMENT OF WHICH INJECTION OF AN AQUEOUS 25 PER CENT. SOL. OF MAGNESIUM SULPHATE WERE MADE IN THE SPINAL SUBARACHNOID SPACE; WITH RECOVERY.

BY AIME PAUL HEINECK, CHICAGO, ILL.

*Professor of Surgery, Reliance Medical College; Adjunct Professor of Surgery, University of Illinois; Surgeon to the Cook Coutny Hospital.*

We used magnesium sulphate, in the method stated above, in our case, and the results were so surprising and so satisfactory that we feel justified in urging its use in tetanus. It is important that the utility and the value of this drug as an agent to control the tonic and clonic muscular contractions so characteristic of this disease be exactly determined. Its value must be decided by the combined experience of clinicians the world over.

Cases of tetanus in the Treatment of which subarachnoid injections of an aqueous solution of magnesium sulphate have been employed:

1. Blake, Jos. A. Male, 15 years, 115 pounds. The use of magnesium sulphate in the production of anaesthesia and in the treatment of tetanus. Surgery, Gynecol. and Obst., Chicago, 1906, vol. II, p. 541.

Period of incubation. Previous immunization. Nature of wound.—Seven days. None. Crushed first three fingers of left hand.

Other Treatment.—Antiseptic disinfection of wound. On 3rd day of disease (10th of injury) 40 cm. of antitetanic serum injected in spinal cord between 4th and 5th cervical vertebrae. 20 c. c. injected in median cephalic vein. On night of same day 20 c. c. injected in media nbasilic vein. On 11th day after injury, 35 c. c. of antitetanic serum injected in spinal canal by lumbar puncture. Chloral hydrate and morphine given when patient not under the effect of magnesium sulphate.

Magnesium Sulphate Treatment.—On the 12th day of injury intra-spinal injection of 4.5 c. c. of magnesium sulphate (25 in 100 of water). 33 hours later repeated injection. 37 1-2 hours later intraspinal injection 8 c. c. of a 12 1-2 per cent. solution of magnesium sulphate. 27 hours later repeated above injection. Six days after repeated same injection.

Result.—Recovery.

Comments.—Injections have a marked effect in restraining the convulsions and relieving pain, thereby conserving strength and preventing excess metabolism and heat production.

2.—Markoe, F. H. Male, 4 years, 40 pounds. Reference same as case 1, p. 549.

Period of incubation. Previous immunization. Nature of wound.—Seven days. None. Sloughing wound of skin and subcutaneous tissue of the right leg.

Other Treatment.—Four injections each of 5 c. c. of antitetanine serum were injected into buttock, the external jugular vein, the spinal canal, and back respectively. Occasional doses of morphine and chloral.

Magnesium Sulphate Treatment.—1.5 c. c. of a 25 per cent solution of magnesium sulphate were slowly injected into the subarachnoid space.

Result.—Died 28 hours after first symptom of disease appeared.

Comments.—Death cannot be attributed in the slighest degree to the magnesium sulphate. On autopsy cultures of tetanus bacillus were obtained from the wound, spleen, and heart blood, showing a marked tetanus bacteriaemia.

3.—Logan, Samuel. Male, 11 years, 80 pounds. The treatment of tetanus by intra-spinal injections of magnesium sulphate for the control of convulsions. Jour. A. M. A. 1906, vol. XLVI, p. 1502.

Period of incubation. Previous immunization. Nature of wound.—Eight days. None. Gunshot wound of hand with old toy pistol loaded with blank cartridge.

Other Treatment.—Simple cleansing of wound after development of the disease. On day of admission 50 c. c. of antitetanic serum injected intra-spinally. Chloral hydrate, gr. 15, sodium bromide, gr. 30, every 4 hours. On 3rd day after admission 10 c. c. antitetanic serum injected in each brachial plexus, in each sciatic nerve, and into the tissues around wound, in all 50 c. c.

Magnesium Sulphate Treatment.—On 3rd day after admission general anaesthesia. 4 c. c. of a 25 per cent. solution of magnesium sulphate injected in spinal canal by lumbar puncture. On 4th day again gave patient general anaesthesia and injection in subarachnoid space by lumbar puncture, 50 minims of 25 per cent. solution magnesium sulphate.

Result.—Death 40 hours and 50 minutes after 1st injection of magnesium sulphate. Heart failed before respirations affected.

Comments.—Temperature 108.2 F. per rectum. Complete cessation of muscular convulsions followed introduction of magnesium sulphate.

4.—Logan, Samuel. Female, 24 years. Reference same as above.

Period of incubation. Previous immunization. Nature of wound.—Seventeen days. None. Vaccination.

Other Treatment.—100 c. c. of antitetanic serum injected subcutaneously. 30 hours after appearance of first symptom, wide excision of vaccination wound, and dusting of surface with dried antitetanine serum.

Magnesium Sulphate Treatment.—30 hours after first symptoms were noticed 4 c. c. of a sterile 25 per cent. solution of magnesium sulphate were injected into spinal subarachnoid space by lumbar puncture. Local anaesthetic employed. 17 1-2 hours later injection was repeated.

Result.—Death 50 hours after appearance of first symptoms.

Comments.—No good resulted from the use of the magnesium sulphate solution. Patient was moribund when 2nd injection of magnesium sulphate was made.

5.—Franke, Morgan. Male, 32 years. Ein Fall von tetanus behandelt mit intra duralem injectionen von magnesium sulphuricum. Zentral. fuer Innere Medicin, 1907, vol. XXVIII, p. 344.

Period of incubation. Previous immunization. Nature of wound.—Twelve days. None. Wound on the middle finger.

Other Treatment.—Energetic antiseptic handling of wound is recommended by this author. Amputation of finger. Chloral hydrate, gr. 30 per rectum daily.

Magnesium Sulphate Treatment.—19 days after infliction of injury, intradural injection of 1 c. c. of sterilized 25 per cent. solution of magnesium sulphate. 5 days after above intradural injection of 2 c. c. of same solution. 4 days later repeated same injection. Injecting needle broke in tissues. Removed by operation.

Result.—Recovery.

Comments.—Franke noticed after each injection of magnesium sulphate that there was a lessening of contracture, also noticed that the injections exerted a beneficial action on the muscular convulsions. Sleep was better. Nourishment possible.

6.—Robinson, Canby G. Male, 11 years, 67 1-2 Pounds. Treatment of tetanus by intraspinal injections of magnesium sulphate. Jour. Am. Med. Assn., 1907, vol. XLIX, p. 493.

Period of incubation. Previous immunization. Nature of wound.—Contusion of scalp. None. Played considerably around stable.

Other Treatment.—Excised supposed wound of entrance.

Chloral hydrate, gr. 30, sodium bromide, gr. 60, every 24 hours for the first two weeks.

Magnesium Sulphate Treatment.—On the 11th day of the disease, patient was anaesthetized. Ethyl chloride used as a general anaesthetic. 3 c. c. of a 25 per cent. solution of magnesium sulphate injected in subarachnoid space. On the next day repeated injection using 3 1-2 c. c. On 15th day of disease injected in same locality 4 c. c. of same solution.

Result.—Recovery.

Comments.—Author states that the intraspinal injections of magnesium sulphate produced marked lessening of the very severe symptoms for a number of hours. The muscular rigidity was never so severe after each injection as it had been before.

7.—Meltzer, S. J., and Auer, Jno. Male, 35 years. The Journal of Experimental Medicine, 1906, vol. VII, p. 705.

Period of incubation. Previous immunization. Nature of wound.—Four days. Insignificant wound of foot which healed rapidly.

Other Treatment.—Large doses of antitetanine serum and sedatives gave no relief. 2 hours before death, an intravenous injection of antitoxine serum was given.

Magnesium Sulphate Treatment.—One intraspinal injection of magnesium sulphate 1 c. c. to every 18 pounds of body weight.

Result.—Death 5 hours after injection of magnesium sulphate solution in subarachnoid space.

Comments.—Anaesthetizing and relaxing effect complete. Respiration good to end.

8.—Miller, Robert. Male, 7 years, 60 pounds. Treatment of tetanus with subarachnoid injections of magnesium sulphate. The Am. Jour. of the Med. Sciences, 1908, vol. CXXXVI, p. 781.

Period of incubation. Previous immunization. Nature of wound.—Seven days. None. Lacerated wound of left hand.

Other Treatment.—Antitoxin daily for 14 doses varying from 1,500 to 7,000 units. Sedatives for a short time. Copious saline enemas and infusion.

Magnesium Sulphate Treatment.—Eleven lumbar punctures made within 13 days. Approximately 25 c. c. of a 25 per cent. solution of magnesium sulphate being injected into the meninges at each puncture.

Result.—Recovery.

Comments.—"Of the value of the treatment by magnesium sulphate, no one who witnessed this case has any doubt." The muscular paralysis following each injection lasted from 18 to 29 hours. It involved all muscles, except those of head, neck and diaphragm. The injections were followed several times by respiratory collapse lasting 11 to 14 hours and the pulse dropped, though not to a dangerous degree.

9.—Henry, Jno. Norman. Male, 9 years. International Clinics, 1908, Series 18, vol. IV, p. 1. Case I.

Period of incubation. Previous immunization. Nature of wound.—Six weeks. None. Abrasion of skin of back by kick of horse.

Magnesium Sulphate Treatment.—Lumbar puncture 3 c. c. of 25 per cent. solution of magnesium sulphate injected in subarachnoid space. 5 days later subarachnoid injection repeated.

Result.—Recovery.

Comments.—The case was a severe one. Made an excellent recovery. Each injection was followed by a relaxation of the rigidity.

Case II.—Male, 19 years, 123 1-2 pounds.

Period of incubation. Previous immunization. Nature of wound.—Seven days. None. Stepped on a nail. At time of admission, the wound was healed.

Other Treatment.—Wound of foot excised.

Magnesium Sulphate Treatment.—Lumbar puncture 6 c. c. of sterile solution of magnesium sulphate injected into spinal canal. Ethyl chloride used as anaesthetic.

Result.—Death admitted July 30th, died August 2nd.

Comments.—One hour after injection patient was entirely relaxed. A rise of temperature followed the intraspinal injection.

Case III.—Male, colored, 9 years, 55 pounds.

Period of incubation. Previous immunization. Nature of wound.—Six days. None. Stepped on nails with both feet and inflicted punctured wounds.

Magnesium Sulphate Treatment.—Lumbar puncture, 4 c. c. of clear spinal fluid withdrawn. 2 1-2 c. c. of 25 per cent. solution magnesium sulphate injected into spinal canal. Two days later repeated injection, only gave 2 c. c. at second injection.

Result.—Death.

Comments.—A rise of temperature followed each injection.

Case IV.—Male, 45 years.

Period of incubation. Previous immunization. Nature of wound.—Three weeks. None. Stepped on nail.

Other Treatment.—On same day as second subarachnoid injection, 18 c. c. of antitetanus serum were given subcutaneously. On the morrow, 30 c. c. of antitetanic serum were injected into the left buttock.

Magnesium Sulphate Treatment.—6 c. c. of 25 per cent. solution of magnesium sulphate injected into subarachnoid space by lumbar puncture. 3 days after above, performed lumbar puncture, removed 35 c. c. of clear spinal fluid, and injected 6 c. c. of solution of magnesium sulphate.

Result.—Death on evening of 2nd day following injection.

Comments.—"It is very much a question whether the magnesium sulphate did not contribute to the patient's death."

1.—Anders, Jas. M. and Morgan, Arth. C. Tetanus, a preliminary report of a statistical study. Journal Am. Med. Assn., 1905, vol. XLV, p. 314.

2.—Anderson, Bruce. "Tetanus following a burn." Australasian Medical Gazette, 1907, vol. XXVI, p. 123.

3.—Vincent, G. Tetanos et Quinine. Annales de l'Institut Pasteur, 1904, No. 12, p. 748.

4.—Simpson, W. J. The evidence and conclusions relating to the Mulkowal tetanus cases. Practitioner, London, 1907, vol. 78, p. 796.

5.—Heddaeus, A. Tetanus nach subkutaner Gelatineinjektion nebst Bemerkungen uber die Anwendung der Gelatine bei Blutungen. Munchen., Med. Wochenschr., 1908, No. 5, p. 231.

Dieulafoy, G. Un cas de tetanos consecutif a une une injection de serum gelatine. Bull de l'Acad. de Med. de Par., 1903, vol. XLIX, p. 901.

6.—McFarland, Joseph. Tetanus and Vaccination; an analytical study of 95 cases of this rare complication. Jour. of Med Research, 1902, vol. VII, p. 474.

6a.—Archibald, E. W. Recent work upon tetanus. Montreal Med. Jour., 1905, vol. XXXIV, p. 874.

6b.—Wilson, Robert. An analysis of 52 cases of tetanus following vaccination. Jour. Amer. Med. Assn., 1902, vol. XXXVIII, p. 1147.

6c.—Churchill, A. H. Tetanus following vaccination. Jour. Am. Med. Assn., 1906, vol. XLVI, p. 1111.

7.—Monro, T. K. Manual of Medicine, p. 166, 2nd edition, London, 1906.

8.—Tourneau. Drei Faelle von Tetanus Deutche Medizinische Wochenschrift, 1904, vol. XXX, p. 347.

9.—Ramsay, A. Maitland. A case of cephalic tetanus following a contusion wound of the outer canthus. Ophthal Record, Chicago, 1904, vol. XIII, p. 537.

10.—Haines, W. D. Tetanus following abortion. Cincinnati Lancet-Clinic, 1904, vol. LII, n.s., p. 48.

11.—Frazier, Charles Harrison. Keen's Surgery, W. B. Saunders Company, 1906, vol. I, p. 47.

12.—Friedlaender, Julius. Fur Lehre vom Rosechen Kopftetanus. Deutsche Med. Wochenschr., 1907, vol. XXXIII, p. 1124.

13.—Anders, J. M. and Morgan, A. C. Tetanus inconatorum. Jour. Am. Med. Assn., 1906, vol. XLVII, p. 2083.

Miron, Georges. Tetanos des nouveaus, nes et son traitement. La Presse medicale, 1905, vol. XIII, p. 708.

14.—Wurdack, Edward. Ueber ein fall von tetanus puerperalis. Prag. Med. Wochenschr., 1903, vol. XXVIII, p. 97.

15.—Zacharias, Paul. Zwei Faelle von Tetanus nach Gynaekologischen Operationen. Muenchen., Med. Wochenschrift, 1908, No. 22, p. 1185.

15a.—Martin, Ed. Post-operative r. tetanus. Zentralbl. f. Gynak., vol. XXX, p. 397.

16.—Vincent, H. Bull. de l'Academie de Med. de Par., 1904, 1907.

16a.—Editorial. The treatment of tetanus. Jour. Am. Med. Assn., June 10, 1905.

17.—Stewart, Jas. American system of practical medicine. Loomis and Thompson. 1897, vol. 1, p. 935.

18.—Demler. Le choix d'un traitement dans le tetanos. Le Progres Med. Par., 1907, vol. XXIII, p. 901.

19.—Symmers, Douglass. The treatment of tetanus by means

23a.—Rogers, Jno. Treatment of tetanus by intraneural and of subcutaneous injections of carbolic acid. Amer. Med., 1903, vol. VI, p. 276.

20.—Morel, C. Un cas de tetanos traite par des injections d'eserine. Gaz. d. hop., 1905, vol. LXXVIII, p. 298.

21.—Binning, Alex. A case of tetanus treated by chloral hydrate; recovery. Brit. Med. Jour., London, 1904, vol. II, p. 1460.

Maberly, John, Tetanus and chloral hydrate. Lancet, London, 1905, Vol. I, p. 1292.

21a.—Jacobson, N. and Pease, H. D. The serum therapy Gegenwart, 1907, vol. XLVIII, p. 49.

23.—Kuester, E. Ueber die Antitoxin-Behandlung des Tetanus, zumalmit intraneuralen Injectionen. Die Therapie der behandelte Falle von Trismus and tetanus. Muenchener Medizin of tetanus. Annals of Surgery., Philadelphia, 1906, vol. XLIV, p. 331.

22.—Weischer, Th. Ueber zwei mit Behring schen-serum 1898, vol. XII, p. 369.
Wochensch., 1897, vol. LXVI, p. 1284.
pie des Tetanus Traumaticus. Muenchen. Med. Wochensch.,

22a.—Heddaeus, A. Ueber den heutigen Stand der Thera-intraspinal injections of antitoxin. Jour. Amer. Med. Assn., 1905, vol. XLV, p. 12.

24.—Hopkins, S. D. Intracerebral injections of antitetanic serum in traumatic tetanus. Med. News, N. Y., 1904, vol. LXXXV, p. 1125.

25.—Moschcowitz, Alexis. Tetanus. Annals of Surgery, 1900, Vol. XXXII, p. 575.

26.—Mornac, G. Traitement du tetanos par les injections epidurales du serum antitetanique. Presse Méd., 1905, ier semestre, vol. VIII, p. 92.

27.—Demoulin, Potherat, Reynier, Rieffel, Delbet, Thiery, Kummer. Bull, et Mem. de la Soc. de Chir. de Par., 1907, vol. XXXIII, p. 383, 424, 431, 451 and 454.

28.—Krummer. Rev. Med. de la Suisse Romande, 1907, vol. XXVII, p. 614.

29.—Scherck, H. J. Antitetanic serum in Fourth of July injuries. Jour. Amer. Med. Assn., Chicago, 1906, vol. XLVII, p. 500.

30.—Dionis De Sejour. Sur la duree de l'immunite donnee par une injection de serum antitetanique. Gaz. d. hop. Paris,

1905, vol. LXXVIII, p. 606. Also Vallas, XIX e Congres de l'Association Francaise de Chirurgie.

31.—Meltzer, S. J. and Auer, Jno. The effects of intraspinal injection of magnesium salts upon tetanus. Jour. of Exper. Med., 1906, vol. VIII, p. 692.

32.—Meltzer and Auer. The toxicity of intravenous injections of magnesium sulphate. Amer. Jour. of Physiol., 1905-06, vol. XV, p. 381.

33.—Miller, Robert T. Treatment of tetanus with subarachnoid injections of magnesium sulphate. The American Jour. of the Med. Sciences, 1908, vol. CXXXVI, p. 781.

34.—Hessert, Wm. Surgery, Gynecology, Obstetrics, 1909, vol. IX.

35.—Lyon, Morton. Jour. Am. Med. Assn., Chicago, 1908, vol. 1, p. 1688.

36.—Greeley, Horace. Magnesium sulphate successful in two cases of tetanus. Jour. Amer. Med. Assn., 1907, vol. XLIX, p. 941.

37.—Cruveilhier, L. Resultats experimentaux concernant l'emploi du sulfate de magnesie dans le traitement du tetanos. Comptes-rendus de la Societe de Biologie, 1908, vol. LXIV, p 111.

